# Providing ‘the bigger picture’: benefits and feasibility of integrating remote monitoring from smartphones into the electronic health record: Findings from the Remote Monitoring of Rheumatoid Arthritis (REMORA) study

**DOI:** 10.1093/rheumatology/kez207

**Published:** 2019-07-23

**Authors:** Lynn Austin, Charlotte A Sharp, Sabine N van der Veer, Matthew Machin, John Humphreys, Peter Mellor, Jill McCarthy, John Ainsworth, Caroline Sanders, William G Dixon

**Affiliations:** 1 National Institute for Health Research Collaboration for Leadership in Applied Health Research and Care, Greater Manchester, Salford Royal NHS Foundation Trust, Salford; 2 National Institute for Health Research School for Primary Care Research, The University of Manchester; 3 Centre for Epidemiology Versus Arthritis, Division of Musculoskeletal and Dermatological Sciences, School of Biological Sciences, The University of Manchester; 4 NIHR Manchester Biomedical Research Centre, Central Manchester University Hospitals NHS Foundation Trust, Manchester Academic Health Science Centre; 5 Alliance Manchester Business School, The University of Manchester; 6 Centre for Health Informatics, Division of Informatics, School of Health Sciences, Faculty of Biology, Medicine and Health, Imaging and Data Sciences, Manchester Academic Health Science Centre, The University of Manchester; 7 National Institute for Health Research Greater Manchester Patient Safety Translational Research Centre, The University of Manchester, Manchester; 8 Rheumatology Department, Salford Royal NHS Foundation Trust, Salford, UK

**Keywords:** RA, remote monitoring, smartphones, patient-generated health data, doctor–patient communication

## Abstract

**Objectives:**

To establish the acceptability and feasibility of collecting daily patient-generated health data (PGHD) using smartphones and integrating PGHD into the electronic health record, using the example of RA.

**Methods:**

The Remote Monitoring of RA smartphone app was co-designed with patients, clinicians and researchers using qualitative semi-structured interviews and focus groups, including selection of question sets for symptoms and disease impact. PGHD were integrated into the electronic health record of one hospital and available in graphical form during consultations. Acceptability and feasibility were assessed with 20 RA patients and two clinicians over 3 months. A qualitative evaluation included semi-structured interviews with patients and clinicians before and after using the app, and audio-recordings of consultations to explore impact on the consultation. PGHD completeness was summarized descriptively, and qualitative data were analysed thematically.

**Results:**

Patients submitted data on a median of 91% days over 3 months. Qualitative analysis generated three themes: RA as an invisible disease; providing the bigger picture of RA; and enabling person-centred consultations. The themes demonstrated that the system helped render patients’ RA more visible by providing the ‘bigger picture’, identifying real-time changes in disease activity and capturing symptoms that would otherwise have been missed. Graphical summaries during consultations enabled a more person-centred approach whereby patients felt better able to participate in consultations and treatment plans.

**Conclusion:**

Remote Monitoring of RA has uniquely integrated daily PGHD from smartphones into the electronic health record. It has delivered proof-of-concept that such integrated remote monitoring systems are feasible and can transform consultations for clinician and patient benefit.

**Rheumatology key messages**

- Daily remote monitoring using a smartphone app was viewed positively by patients and completed regularly.
- Graphs of patients’ daily data identified changes in disease that would otherwise have been missed.
- Patients valued consultations being focused around their own data, supporting person-centred care.

## Introduction

One in four people in the UK live with a long-term condition [1]. Despite accounting for 70% of the National Health Service (NHS) budget, people with long-term conditions spend <1% of their time in contact with healthcare professionals [2]. Patients with long-term conditions thus need to be supported to manage their own health and to make best use of clinical consultations. RA, which affects around 0.7% of the population, is a good example, and people living with RA experience continuous, daily symptoms that fluctuate over time [3, 4]. For healthcare professionals, understanding at a single consultation how symptoms change between visits, often 6 months apart, can be challenging: patients find it difficult to recall and summarize fluctuating symptoms; memory is prone to significant bias [5, 6]; help-seeking behaviours vary between patients [7]; and short consultation times may limit how thoroughly a history is explored. Furthermore, there may be a mismatch between information that clinicians wish to elicit and issues important to patients [8].

Consumer technology provides an unprecedented opportunity to improve collection of patient-generated health data (PGHD) to inform clinical management and self-management of long-term conditions, as well as supporting research [9–12]. The NHS is committed to ‘exploit[ing] the information revolution [and] an expanding set of NHS accredited health apps that patients will be able to use to organize and manage their own health and care’ [2]. Smartphone use continues to increase with over 7 in 10 adults owning a smartphone in England, including 40% of those aged 65–74 and 20% of people aged over 75 [13]. Health apps are widespread, with over 300 000 available [9] including apps specific for RA [14].

At present, however, the opportunities of PGHD are not being fully harnessed and approaches are fragmented. Systems typically support symptom tracking for self-management [14], which are separate from those supporting bespoke research studies [15, 16], while PGHD only occasionally guides clinical care [17]. PGHD have been collected successfully in clinical settings, for example by using tablet computers in outpatient clinics [18, 19]. However, to date, attempts to integrate PGHD data collected outside the clinic context (e.g. at home) into electronic health records (EHRs) have largely failed due to multiple barriers and concerns from patients, clinicians and providers, as well as technical, privacy and governance issues [20, 21]. The electronic exchange of PGHD to clinicians remains restricted to emailing information and early uptake of patient portals tethered to EHRs, with very limited experience of sharing data captured via patients’ mobile devices within clinical systems [21].

The Remote Monitoring of RA (REMORA) study designed and tested a system to support clinical care and research, enabling people living with RA to report daily symptoms using a smartphone app with data integrated into the EHR. The objective of this study was to evaluate the system’s acceptability and feasibility including exploration of participants’ views and experiences of remote monitoring, with specific focus on how integration of smartphone data into the EHR in graphical format influenced consultations.

## Methods

### System development

The REMORA system entailed a smartphone app that enabled patients with RA to monitor their symptoms and impact of disease daily, with the resultant PGHD integrated into a research database and the EHR, providing graphical summaries of longitudinal data during consultations. We followed a stepwise process for system development and evaluation, consisting of three rounds: (i) co-designing and building the prototype app and its integration into the EHR of Salford Royal NHS Foundation Trust; (ii) end-to-end prototype testing for 4 weeks in a small group of patients; and (iii) initial evaluation of the system’s feasibility, acceptability and benefits in a larger group of patients for 85 days. Our six-member patient and public involvement (PPI) group of people with RA met 13 times to feed into development of the REMORA system and study information and outputs.

### Round 1: co-designing and building the system’s prototype

Qualitative semi-structured individual interviews and focus groups with patients recruited from Salford Royal NHS Foundation Trust rheumatology department explored the frequency and characteristics of which electronic patient-reported outcomes might be collected, and why. Clinicians were recruited from the hospital’s rheumatology department, as well as UK researchers with an interest in rheumatology. A table of potential electronic patient-reported outcomes divided into daily, weekly and monthly question sets was refined with input from the PPI group and feedback from subsequent interviews. The study team made the final decision on the question sets, taking into account all stakeholder views. Patients’ views were also sought on their previous experiences of consultations and their baseline views of remote monitoring.

**Fig. 1:**
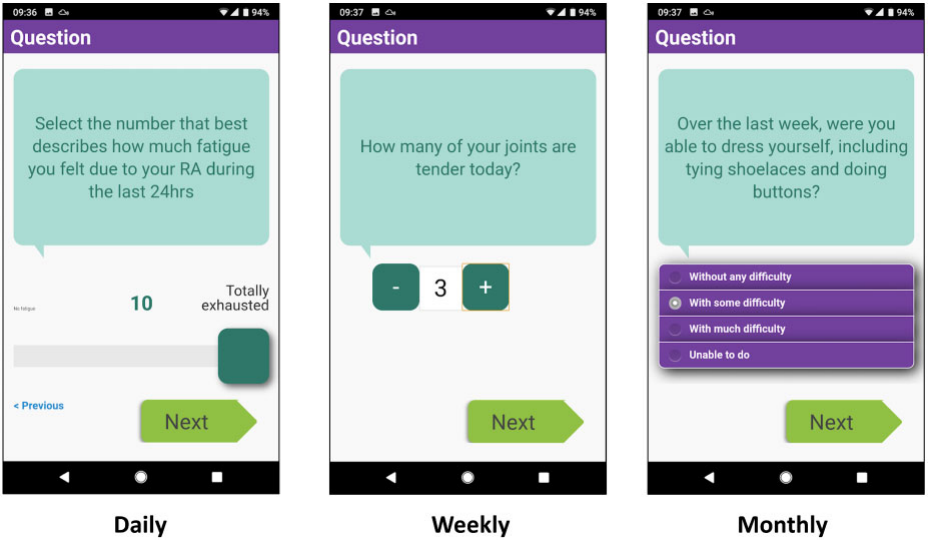
Screen-shots of the Remote Monitoring of RA app functionality including a slider, stepper and radio buttons

**Fig. 2:**
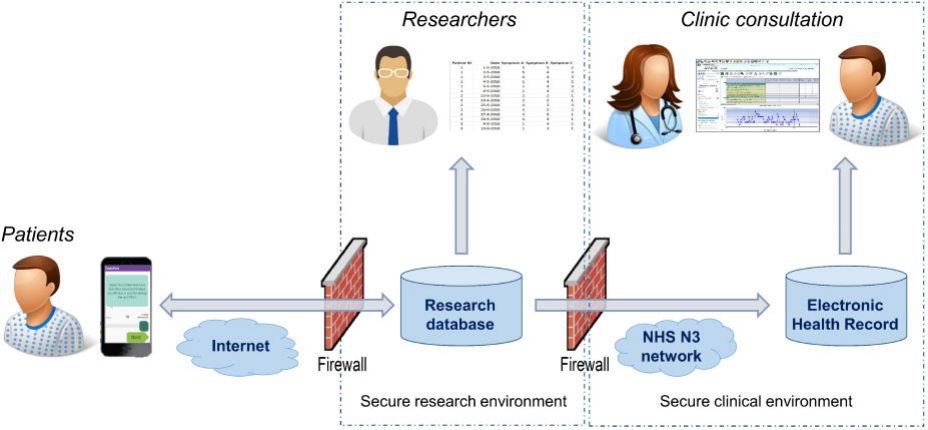
System architecture for integrating data from the smartphone app to the electronic health record

**Fig. 3:**
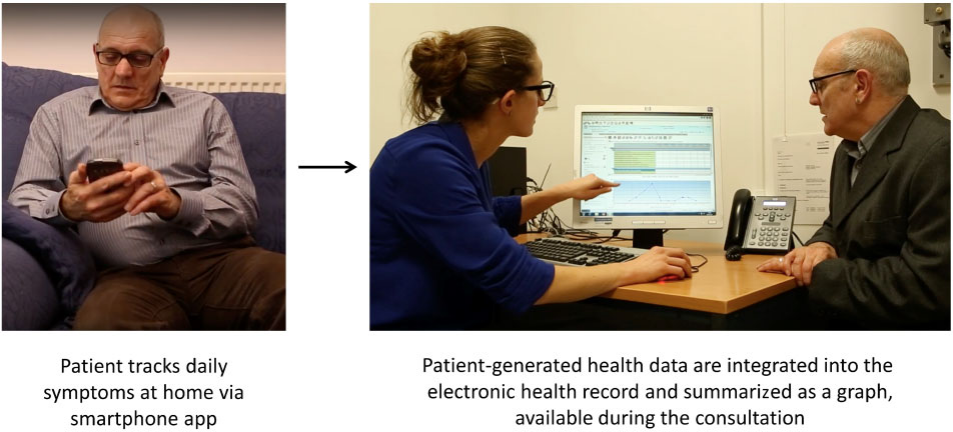
Flow of data from smartphone at home to display in electronic health record within consultation

The app specification was shaped by the contributions of both the stakeholder interviews and PPI meetings. App development by the Connected Health team at the Centre for Health Informatics (University of Manchester) built upon the ClinTouch platform for tracking symptoms in people with serious mental illness [22] and was designed to optimize engagement, ease of use and efficiency. The user interface for ClinTouch was designed by an external design agency. Simple control elements such as sliders, steppers and radio buttons were used to accommodate restrictions in hand function (Fig. 1). A daily alarm prompted reporting at 18:30. Completion of daily scores took around 1 min while a monthly question set could take up to 5 min. The app was suitable for Android phones only. The technical process for EHR integration is summarized in Fig. 2. The patient and clinical experience of data flow is shown in Fig. 3. As the app was developed only for the research study, it is not commercially available although a demonstration version is available by request from the authors.

### Data items

The following items were included within the app (Table 1). Daily: all items from the RA Impact of Disease questionnaire adapted for daily use [23] and one item on morning stiffness. Weekly: self-reported tender and swollen joint counts from the Disease Activity Score 28 [24]; two items on the occurrence of flares; a global assessment of disease activity [25]; and the Work Productivity and Activity Impairment questionnaire [26]. Monthly: the Health Assessment Questionnaire [27].

**Table 1:** Question sets related to disease activity and impact, and their frequencies

Data item	Question stem	Scale	Anchors
Daily data collection			
Pain	Select the number that best describes the pain you felt due to your RA during the last 24 h	VAS	None (0); extreme (10)
Function	Select the number that best describes the difficulty you had in doing daily physical activities due to your RA during the last 24 h	VAS	No difficulty (0); extreme difficulty (10)
Fatigue	Select the number that best describes how much fatigue you felt due to your RA during the last 24 h	VAS	No fatigue (0); totally exhausted (10)
Sleep	Select the number that best describes the sleep difficulties (i.e. resting at night) you felt due to your RA during the last 24 h	VAS	No difficulty (0); extreme difficulty (10)
Physical well-being	Considering your arthritis overall, how would you rate your level of physical well-being during the last 24 h?	VAS	Very good (0); very bad (10)
Emotional well-being	Considering your arthritis overall, how would you rate your level of emotional well-being during the last 24 h	VAS	Very good (0); very bad (10)
Coping	Considering your arthritis overall, how well did you cope (manage, deal, make do) with your RA during the last 24 h?	VAS	Very well (0); very poorly (10)
Morning stiffness	How long did your morning stiffness last today? (please enter ‘0’ if you did not experience any stiffness)	7-point Likert scale	0 min; 0–9 min; 10–19 min; 20–29 min; 30–59 min; 1–2 h; >2 h
Weekly data collection			
Tender joint count	How many of your joints are tender today?	NRS	0 to 28
Swollen joint count	How many of your joints are swollen today?	NRS	0 to 28
Patient global assessment	Considering all of the ways your arthritis has affected you, how do you feel your arthritis has been in the last week?	VAS	Very well (0); very poor (100)
Employment status	Are you currently employed (working for pay)?	Dichotomous	Yes; no
Hours missed due to health problems	During the past seven days, how many hours did you miss from work because of problems associated with your RA?	n.a.	0 to [no upper limit]
Hours missed due to other reasons	During the past seven days, how many hours did you miss from work because of any other reason, such as vacation, holidays, time off to participate in this study?	n.a.	0 to [no upper limit]
Hours actually worked	During the past seven days, how many hours did you actually work?	n.a.	0 to [no upper limit]
Degree health affected work productivity	During the past seven days, how much did your RA affect your productivity while you were working?	VAS	RA had no effect on work (0); RA completely prevented me from working (10)
Degree health affected daily activities	During the past seven days, how much did your RA affect your ability to do your regular daily activities, other than work at a job?	VAS	RA had no effect on my daily activities (0); RA completely prevented me from doing my daily activities (10)
Occurrence of flare	Have you experienced a flare in the last week?	Dichotomous	Yes; no
Flare description	Please describe how your flare has affected you	Free-text^a^	n.a.
Health Assessment Questionnaire	Validated questionnaire consisting of 23 items to assess physical function in RA, including items related to the usual ability to perform a range of activities (e.g. get in and out of bed, take a bath) over the last week	n.a.(overall calculated score 0–3)	n.a.

a For people reporting a flare, there was a free text field to enter information on its impact and potential causes. The app also had a diary function for patients to record symptoms, feelings and thoughts in free text to support self-management and add context to support discussions in clinic. n.a.: not applicable; NRS: numerical rating scale; VAS: visual analogue scale.

The app also had a free-text diary to record notes to support self-management and clinic discussions: these data were not incorporated into the EHR. Additional items were suggested (e.g. diet and exercise) but not included because of the perceived burden of data collection, lack of consensus on their importance and/or availability of validated instruments.

### Round 2: testing the prototype

Round 2 tested the prototype to identify problems and refine the design. RA patients were invited to record their symptoms for 4 weeks. Following a 30-min baseline clinical consultation, participants received face-to-face instructions on how to use the app. Informed by the PPI group and a nurse specialist, instructions included how to perform tender and swollen joint count self-assessment [28]. Since PGHD were not monitored between consultations, patients were advised to take usual action in case of health problems. Members of the research team set up patients’ phones and supported them throughout the study. Phones with the app pre-loaded were available on loan for people without an Android phone. Patients did not receive any financial compensation for their participation.

After 4 weeks, patients reviewed symptoms with a rheumatologist during a 30-min research consultation. They participated in an interview about their experience of using the app. Consultations were audio-recorded and transcribed for patients who provided consent. We summarized key feedback on the prototype that, together with identified technical issues, informed further development into the final system.

### Round 3: evaluating system acceptability and feasibility

In Round 3 we explored the acceptability, feasibility, benefits and limitations of using the REMORA system for 85 days. We aimed to recruit up to 30 patients (shaped by previous experience of sample size sufficient to achieve saturation, and pragmatic factors). Recruitment, consenting, instruction, support and follow-up of patients were similar to Round 2. Two clinicians conducted the consultations, both of whom were interviewed to capture their views on the usefulness of app data during consultations.

### Analysis of feasibility, acceptability and perceived benefits and limitations

Interview and consultation data were analysed thematically, drawing on some of the key techniques of grounded theory [29], including open coding, constant comparison and memo-writing. Initial interviews were structured by key topics of interest; however, questions were framed to facilitate exploratory discussions on both positive and negative aspects of expectations and experience, as well as the history and context of experience (the interview guide is included as Supplementary Material available at *Rheumatology* online). Analysis was iterative and reflected deductive and inductive aspects to developing analysis with emerging issues further explored in subsequent interviews [30]. The primary coding of qualitative data was conducted by L.A. (organized using NVivo qualitative data analysis software; QSR International Pty Ltd, Version 11, 2015) who also conducted the interviews. Initial coding formed the basis for long descriptive accounts of the coded data that were circulated and discussed and refined initially in analysis meetings with C.S. (as supervisor and lead co-investigator for qualitative research). The summaries and coding were discussed at wider team meetings and via comments from clinical researchers (W.D. and C.A.S.). Initial codes were grouped to form three core thematic categories based on multiple sources of interview data along with the recorded clinical observations.

Quantitative evaluation analysed the completeness of submitted app data. Per participant, there were a maximum of 85 daily, 13 weekly and 3 monthly opportunities to enter data. The number of active days was calculated as the number of days between the first and last day of submitting daily scores. Across participants, we calculated the median and interquartile range for the number of daily, weekly and monthly entries, and evaluated missing data. Due to small numbers, we could not analyse factors that influenced patterns of engagement.

### Ethics approval

The Greater Manchester Central Research Ethics Committee approved the study (ref. 15/NW/0172).

## Results

We recruited 26, 8 and 20 patients with RA for Rounds 1, 2 and 3, respectively; seven participated in all three rounds. Ages ranged from 32 to 84 years, with time since diagnosis from <1 year to >30 years. Three-quarters of participants were female. For Round 1, we recruited 10 rheumatology clinicians and 13 researchers. For Round 3, recording failed for one follow-up patient interview and two patients declined consent for the audio-recording, resulting in 17 transcripts.

### Acceptability, feasibility, benefits and limitations

**Fig. 4:**
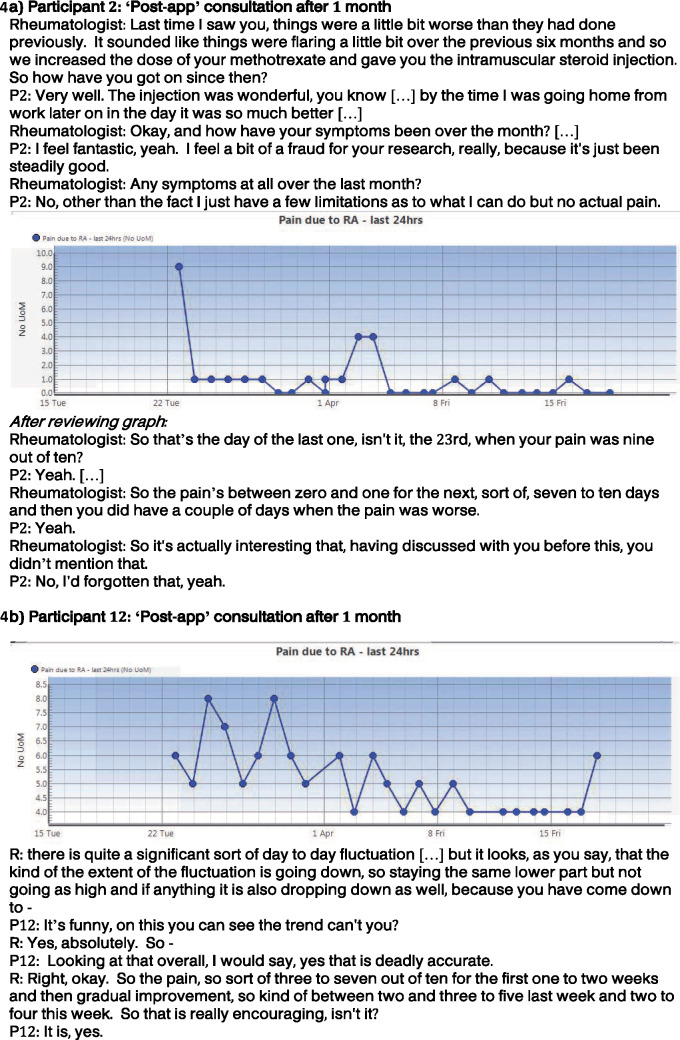
Extracts and graphs from Round 2 clinical consultations from participants P2 (**A**) and P12 (**B**)

Patients found remote monitoring acceptable, with most patients enjoying using the app and finding it easy to use. Daily data collection fitted people’s usual routines, and mostly occurred in the evening following the reminder (quote 1, Table 2). iPhone users who borrowed an Android phone were less able to incorporate REMORA into their usual routine. Although some patients used help from family members in learning how to record data (quote 2, Table 2), or used a stylus, all were ultimately able to use the app independently. For the majority of questions, the frequency seemed appropriate: the main exception was assessment of joints, which some patients felt should be daily instead of weekly. In contrast, there was recognition that people with well-controlled disease could become frustrated with daily reporting in the absence of symptoms. This was reflected in some initial discussion of expectations where participants said they would only enter data on ‘bad days’ (quote 3, Table 2) and suggested there might be need for tailoring the frequency if disease was well controlled (quote 4, Table 2). However, during follow-up interviews after using the app, these respondents talked about ease of use (quote 2, Table 2) and the value of the regularly recorded data for the consultation (see quotes 11, 12 and 14, Table 2; and Fig. 4 for P2) as presented in main themes below.

**Table 2:** Quotes from patients’ and doctors’ views of REMORA

Quote no.	Quote
Acceptability and feasibility	
1	I’m a bit of a 6:30 fan. I get a reminder at 6:30 and I try and do it then because I know what my memory’s like … I like that [reminder] because I know myself I’ve got to do it as soon as I get that reminder, if I’m able to. I have to get it done then because I do tend to get into something else and then I completely forget. [P6]
2	It’s good, alright, it’s fine … The first few days, yeah, I couldn’t—I kept forgetting how to get onto the app but no, yeah, it’s a doddle … my grandson came and he showed me what to do. But I’ve done it greatly since. I’ve not forgotten how, you know. I remembered what he taught me and I did it and yeah, and it’s good. [P10]
3	I would do it on my bad days, I probably wouldn't on my good days. But I take methotrexate and I take that weekly. I could discuss my week with an app when I'm taking my tablets. [P2]
4	Everything seems to have just calmed down … it took about 3 weeks or so and then all of a sudden just nothing at all; so I suppose from my perspective, with putting all the readings at zeros, it became a bit laborious … just that because some of them [questions] are there every day, it’s very repetitive, ‘And how are you feeling today?’ and ‘Are you still in a job?’ … ‘[I’ve] told you once’. [P1]
Providing the ‘bigger picture’ of RA	
5	The other night my wrist … was so bad I could have chopped that arm off … I went to get the app because I thought, I want to put it on. It was just a natural thing, I need to put this down, I need to make this—you know, so I can remember to tell the consultant how I felt right now. [P15]
6	It's difficult because sometimes you're fine and you have to go and see the doctor, and the doctor asks all these questions, but this [app] sort of builds a picture in that respect. [P18]
7	He said to me, ‘How are you?’ like that particular day and I was fine … And he was able to say to me, well, you weren’t so good, you had a bit of a blip on such and such a day … I’d forgotten about that [at previous visit] I had the steroid injection and I felt so much better, more or less immediately … But yeah, I’d still had a bit of a blip in between all that and he was able to see that. [P2] (Fig. 4A)
8	The graphic was perfect … you could see the trend, which I found very encouraging because up until then I thought these biologics are expensive, why aren’t they working? … It’s only really when I saw that graph that they were giving more than I realized. [P12] (Fig. 4B)
9	There’s the ability to see the impact of interventions and see the rapidity of change. There was the ability to see gradual trends in disease severity within day-to-day fluctuations that might otherwise obscure that gradual trend. [D2] (Fig. 4B)
10	So you say to patients, Oh, how have you been? I've been fine. Have you had any flares? No. Okay, great. Well, just carry on then because you believe them. But in this case, somebody might say the same things and I'd say, Well actually, your chart on pain says you had a big blip here. Look at it. And they say, Oh yes. And then you'd say, Well, did something … can you remember that? Did something happen? And then they'd say, Oh yes. [D1]
Enabling patient-centred consultations	
11	It made a difference, because it wasn’t all me telling him and trying to remember, the information was there, so you’ve got solid proof straightaway. [P1]
12	Sometimes you do feel as if you’re just moaning all the time, I’m in pain, or whatever. But if it’s there and he can see it on the screen, it’s like it’s said it for you. [P2]
13	I think it makes it more personal to you. Because then [the rheumatologist] is looking on the app as how I’ve felt, how I’ve interpreted my rheumatoid … So yeah, I think it would give you a little bit more confidence too … So to see it on the screen over the 6 months, then I think you’d feel better knowing that they’re looking at you, rather than what they’ve wrote about you [previously]. [P14]
14	How can you talk about something when there’s missing information? So if you’ve got that graph, there’s your information, you’ve got your information, he’s got his information … so when it’s there it’s a shared conversation between us … rather than him asking me questions and me trying to answer them. [P9]
15	Where the app data did uncover different patterns, we were then able to discuss using that visual aid of the graph. And that then supporting people to remember what had happened and being able to explain in more detail. [D2]
16	I knew that we don't address fatigue as much as they would like us to, and that's partly because there's very little to do about it. And as medics we like to fix things and you can't fix it. But actually, I will in future. [D1]
17	I don’t know about [the original rheumatologist] because I think he was old school … Yeah, I don’t think he would have appreciated a mobile phone. However, I think at [a different hospital] when I used to go in … I think we could have talked about that [app data] and then talked about what had gone on over 3 months. [P15]
18	I didn’t see [the research rheumatologist] in a strictly medical context … with [the usual rheumatologists] … it was a different type of consultation … I mean I felt much more at ease with [the research rheumatologist] … not that there’s any problem with [the usual rheumatologist]. [P16]

D: doctor; P: patient; REMORA: Remote Monitoring of RA.

In addition to comments on acceptability and feasibility of the app summarized above (and section 1 of Table 2), analysis of data from patient interviews identified three main themes: (i) RA as an ‘invisible disease’, highlighting that previous experiences of clinical consultations and life with RA rendered symptoms and personal experience as invisible; (ii) providing the ‘bigger picture’ of RA, referring to how graphical representations of data were used within consultations; and (iii) enabling person-centred consultations, reflecting changes in the quality of the consultation and aiding more shared discussions around treatment and management decisions. The latter two themes were also present in interviews with clinicians. As the first theme was largely confirmatory and in line with previous qualitative research on experiences of RA [31–34], we summarize this briefly before reporting on the remaining themes in more depth.

#### RA as an ‘invisible disease’

Patients frequently referred to the ‘hidden’ nature of their symptoms. While the absence of visual signs of RA was undoubtedly frustrating for many participants during their day-to-day lives, it was particularly problematic during rheumatology consultations. The fleeting nature of the disease meant it was not unusual for symptoms to be absent when seen by the rheumatologist. As one respondent stated, ‘I don’t think in that half hour that you’ve got that consultation doesn’t always show the bigger picture of what you’re actually dealing with’*.* REMORA was able to bring the self-management of daily life into the consultation, making patients feel supported in their self-management:

When you explain and their faces don’t look as though it was bad and you think, was it really that bad? [With app data, a practitioner might say] I can see you’ve had this, this and this, we’ll class that as one flare up … And then you'd have an understanding of yourself what a flare up would be. [P14]

As presented in the next two themes, the main value of REMORA was its ability to provide insight into the ‘bigger picture’ of everyday experience of symptoms for both patients and clinicians. These shared insights impacted upon consultations, creating interactions that were more shared, and highlighting issues that might otherwise have been missed.

#### Providing the ‘bigger picture’ of RA

Participants described the benefit of remote monitoring as a way of accurately recording fluctuating symptoms:

You just live with it, so it’s when something different happens, you just think … it would be useful to log that really, because then when you go [to an appointment] you can at least say, well, it happened at this time and it happened at that time. [P7]

The integration of data into the EHR with graphical summaries viewed during consultations was perceived to have a positive impact by patients and clinicians. The frequency of the data ensured fleeting symptoms were captured, with the graph providing an understandable visual summary (quotes 5 and 6, Table 2). Importantly, there were occasions when the graphs picked up flares that patients had forgotten. Additionally, the graphs demonstrated the effectiveness of treatment interventions (both pharmaceutical and therapeutic). These two key benefits are illustrated by quotations from patients and doctors during Round 2 (quotes 7–10, Table 2) and the corresponding graphs and extracts from their recorded consultations (Fig. 4). In both cases, the graphs, covering just 1 month, demonstrated treatment response and the time course of that response. Such variations were described by participants prior to app use as being hidden within their everyday lives, and were considered particularly problematic when trying to convey the changing nature and severity of symptoms within consultations. The examples demonstrate the benefit of the app in drawing an accurate picture that depicts the ups and downs of symptoms over time.

#### Enabling person-centred consultations

The graphical representation of data served as a material prompt to enable person-centred consultations by bringing evidence to frame discussions about changing symptoms, in a way that seemed to transform the nature of interactions within consultations. The data display, plotting the everyday ups and downs, made it easier for patients to play a more active role in discussions and be better heard as, in effect, the data ‘says it for you’ and ‘provides evidence’ of what has been happening (quotes 11 and 12, Table 2). Consequently, patients felt more confident in participating in consultations as the data legitimized their experiences and provided a platform for them to be discussed. Patients felt more able to discuss all symptoms that were collected as part of the daily question set, such as sleep, fatigue and coping, and which they might otherwise find difficult to raise, while physicians were prompted to address these by virtue of their presence on the graphs. Assessment of disease was based more accurately on patients’ experience through time, thereby lending itself to a more accurate and personalized approach to discussions around disease management (quotes 13 and 14, Table 2). Clinicians reported that interpreting the longitudinal REMORA data during consultations was quick and easy, particularly using the graph function. This was considered potentially time-saving when balanced against extracting information through history taking. While not intended as a replacement for history taking, it was felt to provide an efficient starting point for discussion (quote 15, Table 2). Collecting patient-generated data items such as sleep, fatigue and coping also led to discussions that might otherwise not have occurred, seen as beneficial by patients and which influenced future consultations for the clinicians (quote 16, Table 2). Despite the undoubted benefits, patients raised concerns about how the app might be received in routine practice, given that there would likely be practitioner variation in receptivity (quote 17, Table 2). One patient also noted that the consultations with the research rheumatologist had a different feel from their usual consultations (quote 18, Table 2).

### Data completeness

The median number of active days in Round 3 was 82 (interquartile range 80–82). Patients submitted daily scores on almost all possible days (median 91% of days; interquartile range 78–95%), with only 4 of 20 patients submitting data on <60% of days. Patients recorded a median of 73/85 daily (86%) and 11/13 weekly (85%) entries. Monthly HAQ scores were provided only once by 8/20 people, while a further 9/20 and 3/20 people did this for two and for all three months, respectively. Of all 1325 daily question sets, <1% had missing values. Of all 213 weekly entries, 15 (7%) had missing values. All submitted monthly entries were complete.

## Discussion

This study has demonstrated the acceptability and feasibility of using the REMORA system to collect daily symptoms with high levels of completeness over 3 months. It has provided evidence of the perceived benefits of integrating PGHD collected from smartphones into the EHR. Specifically, it demonstrated that patients benefitted from consultations being focused around their own data, making consultations more personal. Furthermore, the summary of daily symptoms revealed disease patterns that would have been missed, including flares and long-term trends that would otherwise be hidden within the day-to-day fluctuation of symptoms.

In keeping with previous studies, patients described their RA as invisible [35], which has been found to lead patients to under-report symptoms [31, 32, 36], or to feel the legitimacy of their symptoms is undermined [34, 37]. This study demonstrates that the integration of PGHD with the EHR enabled greater information exchange and discussion to make illness experience more visible. The graphical representation of data was especially valued, providing the ‘bigger picture’ of disease activity over preceding months, enabling patients and clinicians to visualize temporal symptom changes and add more detail to verbal accounts*.* This capture and visualization of PGHD impacted on the nature of the interaction, validating patients’ role in discussions and initiating conversations that would otherwise not have happened. Patients felt empowered as the data ‘says it for you’, in keeping with previous work that found that visual representations of ‘real time’ information helped ‘get patients on board’ and facilitated discussion of disease management [38]. Ultimately, the data fostered what could be viewed as a more person-centred approach to consultations, prioritizing patient perspectives and promoting more tailored care [39]. Patients deemed the data useful for self-management because it provided a detailed live record of symptoms that previously remained difficult to recall. The longitudinal symptom data in combination with flare reports and diary entries enabled patients to better understand triggers and patterns of symptoms.

From the clinician’s perspective, the REMORA system filled gaps in the usual management of RA by generating a clear picture of symptom progression through time: an acknowledged requirement for providing quality care in arthritis [40]. Such benefits have previously been suggested but seldom proven [17]. PGHD has the potential to positively influence clinical consultations by providing a more accurate picture of changing disease and treatment response; facilitating conversations about topics that may have previously remained hidden [41]; and supporting shared decision making. In addition, the collection of data items such as sleep, fatigue and coping facilitated discussions that might otherwise not have occurred, with positive reflections from both clinicians and patients.

To date, there are few examples of PGHD being integrated successfully into the EHR from home in any disease setting, despite wide acknowledgement of potential benefits [42]. For example, none of the current apps in the NHS apps library (https://apps.beta.nhs.uk/) integrates patient-generated data into EHR systems, making the learning from REMORA informative across a range of long-term conditions. This lack of integration can be explained by many potential challenges and concerns, including technical, privacy and security issues; fears from clinical teams about time pressures and handling increased volumes of PGHD; unproven clinical benefits; and managing change in traditional clinical workflows [20, 43]. For PGHD to be useful in a clinical setting and to minimize workload, it should be easily accessible [11]. By incorporating data into the EHR, being explicit that data would not be viewed between consultations and using existing, trusted graphing functionality, REMORA provided minimal additional burden and no switching between systems.

This study is a significant first step towards demonstrating the benefits of integrating remote monitoring of PGHD into clinical care. However, several important limitations should be acknowledged. This feasibility study was conducted by a small group of enthusiastic self-selected patients and clinicians. The acceptability of such a system to early and late adopters of technology, with different levels of digital literacy, is unknown. The two clinicians conducting the study consultations were part of the research team, which undoubtedly influenced their view of the potential of remote monitoring. Nonetheless, the positive experience of the consultations surpassed their expectations, reinforcing those beliefs and unveiling unforeseen benefits. More sceptical colleagues might have a different experience, as one patient participant suspected (quote 17). Patient engagement was strong throughout the 3 months, but it is unknown to what extent patients would continue to enter daily data over longer periods of time. Furthermore, it is possible that long-term daily tracking and increased screen time may have negative implications not picked up in this study.

There are a number of infrastructure developments required to make remote monitoring scalable for routine practice: in our study, the research team supported the process of downloading the app, linking it to the correct patient’s EHR and taking consent. Clinicians would need to be able to ‘prescribe’ remote monitoring, and patients would need to download the app independently onto any operating system (e.g. Android or iOS), complete user authentication to link to the correct EHR ID and provide digital consent, with data then flowing into a PGHD repository with interoperability to any EHR system and research database. The evidence as to whether remote monitoring provides value for money needs to be established, as does the financial route for sustaining any necessary infrastructure, maintenance and user support. This strong proof of principle study, however, allows this development to proceed in the knowledge that regular, accessible PGHD is potentially transformative for clinical care.

### Conclusion

The REMORA feasibility and acceptability study has provided proof of principle that daily recording of symptoms with integration into the EHR is feasible and viewed positively by people with RA and their clinicians, with high engagement over 3 months. This opens significant opportunities for the transformation of clinical care and research in long-term conditions within the new digital era.

## Supplementary Material

kez207_Supplementary_Data
