# Supplementary material for: Providing ‘the bigger picture’: benefits and feasibility of integrating remote monitoring from smartphones into the electronic health record: Findings from the Remote Monitoring of Rheumatoid Arthritis (REMORA) study
Source: Rheumatology (Oxford). 2019 Jul 23;59(2):367–78. doi: 10.1093/rheumatology/kez207 (PMC7223265; doi:10.1093/rheumatology/kez207)
Supplement: kez207_Supplementary_Data [file kez207_supplementary_data.docx]

**SUPPLEMENTARY MATERIAL**

**REMORA STUDY**

Topic guide for focus group with patients Version 1

Please note: this schedule will be used as a guideline of the areas to be covered. Responses can be probed and followed up, and the schedule will be kept under review as the focus group progresses. This is may be needed to condense some areas to ensure the essential opinions on the app demonstration are covered or expand some areas of relevance to the study.

***Introduction***

- Thank you for agreeing to take part
- Introduction to researchers
- Explanation of research & aims
- Details of participation
- Voluntary (can withdraw any time) and confidential
- Audio recording and data protection
- You do not have to discuss anything that will make you feel uncomfortable
- We would like to hear everybody’s views
- Want to hear a full range of views; no right or wrong answers
- Any questions?
- Ask each participant to introduce themselves, by saying their name, whether they use a mobile phone and if it is a smartphone do they use any apps
- Health economics questionnaire (EQ-5D-5L)

## Living with rheumatoid arthritis and self-management

- What is it like to live with rheumatoid arthritis?
- What resources do you use other than health services for managing your arthritis?
- What is most important to you in terms of support or help with your arthritis?

## Interactions with professionals

- What kinds of things are most important to you when you consult health professionals about management of your arthritis (communication, confidence in knowledge, information, advice etc)?

***Monitoring of Symptoms***

- What symptoms do you think have the most impact on how well or unwell you feel?
- What kind of changes do you watch out for and take as a signal that your condition may be worsening?
- Would you say you actively monitor and/ or record changes in your symptoms?
- Do you find it easy to discuss how your symptoms have been in appointments with health professionals?
- Do you think the healthcare professionals are able to understand the timing, severity and impact of your symptoms based on your description?

*Protocol: The final dataset will be agreed during this process, likely to include numerical scores for disease severity, pain, fatigue, activity and disability with the ability for patients to keep notes in a diary function. ”*

- What questions should be asked to understand how you are feeling? E.g. how is your pain today?
- Do you find it easy or difficult to rate your symptoms on a scale? E.g. on a scale of 1-5 how is your pain today?

## Views and experiences of technologies to assist in managing condition

*Protocol: “This qualitative research will understand the feasibility and acceptability of remote data collection, including an understanding of smartphone ownership and e-literacy, and the motivators and barriers for ongoing patient engagement using smartphones”.*

- Do you currently use a smartphone, tablet or computer?
- How often do you use these and for what type of things (e.g. email, shopping, social networking)
- If not, do you have any concerns about using a smartphone?
- Have you ever used the internet to find information or support in relation to your rheumatoid arthritis? – has this been helpful?
- Have you any experience of using other health related apps? If so, can you give examples and describe how you found using them?

***Views and opinions on using a mobile phone to collect health data and the potential value***

*Protocol: “This qualitative research will understand the feasibility and acceptability of remote data collection, including an understanding of smartphone ownership and e-literacy, and the motivators and barriers for ongoing patient engagement using smartphones”.*

- What are your views about storing and sharing health data collected by monitoring symptoms etc by mobile phone?
- What are your views about collecting information about peoples’ position and movements to monitor physical activity and functioning?
- Do you have any concerns about collecting health data by mobile phone?
- What do you think are the benefits of this type of monitoring?
- Would you be happy for information collected in this way to be used for research about RA?

***Demonstration of initial ideas for the app***

*Protocol: “A mobile phone and the prototype app will be demonstrated at the interview or focus group to explore potential functionality of the system, plus format and design of the user interface. This qualitative research will understand the feasibility and acceptability of remote data collection…. The acceptable frequency of data collection and timing of reminders will be explored. The final dataset will be agreed during this process, likely to include numerical scores for disease severity, pain, fatigue, activity and disability with the ability for patients to keep notes in a diary function. ”.*

- Slides to show initial questions and scoring system
  - - - What are your views about the questions (wording and scoring)?
      - Are any questions problematic?
      - Are there other questions that should be asked?
- Show images of potential interface designs (from our previous apps – uMotif/ ClinTouch) and allow respondent to try using demonstrator versions on mobile phones
- What aspects of the app design do you like or not like and why?
- Demonstrate additional components and features e.g. home screen, information, medication reminders, daily diary
  - - - What features do you like or not like and why?
      - Do you think you would use all of these?
    - Demonstrate versions of feedback reports to summarise their scores
- Which graphs/ summaries are most useful and why?
  - - General use
- How do you think you will find using this?
- How often would you be happy to complete the app data ?
- Do you anticipate any problems using the app?
- Do you have any other comments or questions?

# THANK YOU FOR GIVING YOUR TIME TO DISCUSS THESE ISSUES
